# Neglog: Homology-Based Negative Data Sampling Method for Genome-Scale Reconstruction of Human Protein–Protein Interaction Networks

**DOI:** 10.3390/ijms20205075

**Published:** 2019-10-12

**Authors:** Suyu Mei, Kun Zhang

**Affiliations:** 1 Software College, Shenyang Normal University, Shenyang 110034, China; 2 Bioinformatics Facility of Xavier NIH RCMI Cancer Research Center, Department of Computer Science, Xavier University of Louisiana, New Orleans, LA 70125, USA

**Keywords:** protein–protein interaction, paralog/ortholog, negative data sampling, machine learning, l_2_-regularized logistic regression

## Abstract

Rapid reconstruction of genome-scale protein–protein interaction (PPI) networks is instrumental in understanding the cellular processes and disease pathogenesis and drug reactions. However, lack of experimentally verified negative data (i.e., pairs of proteins that do not interact) is still a major issue that needs to be properly addressed in computational modeling. In this study, we take advantage of the very limited experimentally verified negative data from Negatome to infer more negative data for computational modeling. We assume that the paralogs or orthologs of two non-interacting proteins also do not interact with high probability. We coin an assumption as “Neglog” this assumption is to some extent supported by paralogous/orthologous structure conservation. To reduce the risk of bias toward the negative data from Negatome, we combine Neglog with less biased random sampling according to a certain ratio to construct training data. L_2_-regularized logistic regression is used as the base classifier to counteract noise and train on a large dataset. Computational results show that the proposed Neglog method outperforms pure random sampling method with sound biological interpretability. In addition, we find that independent test on negative data is indispensable for bias control, which is usually neglected by existing studies. Lastly, we use the Neglog method to validate the PPIs in STRING, which are supported by gene ontology (GO) enrichment analyses.

## 1. Introduction

Protein–protein interaction (PPI) is one of the central research topics in experimental and computational biology. Rapid reconstruction of genome-scale protein–protein interaction (PPI) networks is instrumental in not only understanding cellular processes and disease pathogenesis but also developing therapeutic drugs. Recent years have witnessed the rapid accumulation of PPI data in various databases, e.g., HPRD [1], BioGrid [2], Reactome [3], KEGG [4], IntAct [5], HitPredict [6], STRING [7], DIP [8], BIND [9], etc. These databases provide abundant information for us to further experimentally or theoretically analyze the underlying PPI, signaling or other molecular mechanisms [10,11]. The PPI experimental techniques, including X-ray crystallography, yeast two-hybrid, mass spectrometry, and affinity purification, are very credible in general. However, these techniques also exhibit a high fraction of false positive rate and low agreements with each other [12]. To date, various computational methods have been proposed to eliminate these discrepancies and enhance the known PPI databases. Although much effort has been devoted to computational reconstruction of intra-species [13,14,15,16,17,18] and inter-species [19,20,21,22,23] PPI networks, there still are several major issues that need to be properly addressed.

*Data quality* is the first critical factor that affects the performance of computational methods. Because of the limitations of experimental techniques [12], the major PPI databases [1,2,3,4,5,6,7,8,9] more or less contain a certain level of noise. Furthermore, only a small portion of data are experimentally verified physical PPIs [1,2,5]. For instance, the PPI database STRING [7] has collected massive PPI networks of 2031 species, but the data have been reported to be of low quality the majority of which have not been experimentally or computationally validated [23,24]. In general, the PPIs verified by multiple experimental techniques and computational methods are assumed to be of high quality. Meanwhile, how to generate quality negative training data should also attract more attention to train a less biased model, because the experimentally verified negative dataset is very small. Unfortunately, this problem has not been satisfactorily addressed yet.

As regards inter-species or pathogen-host protein interactions, the issue of data scarcity seems to be more serious. In such cases, advanced machine learning approaches such as transfer learning become the first solution to augment training data or borrow useful information from auxiliary data [19,20,21]. However, a large genome gap between source species and target species potentially results in negative knowledge transfer that would adversely affect the model performance [19,25].

*Feature construction* is the second critical factor that determines whether a predictive model could generalize well to unseen examples or patterns. Many features have been used to predict PPIs, e.g., *k*-mer, sequence similarity, binding motif, domain co-occurrence, gene expression profile, gene co-expression, structural similarity, post-translational modification, PPI network topological properties, etc. Among these features, gene ontology (GO) has been reported to be the most discriminative feature [26,27]. As a cheap and easily available feature, sequence *k*-mer is frequently used to predict PPIs [13,15,28]. However, some studies claim that simple sequence *k*-mer cannot predict PPIs [14]. In response to this controversy, Park et al. [15] argues that the failure of computational modeling results from the sampling ratio of negative data instead of the sequence *k*-mer feature itself.

*Negative data sampling* is the third critical factor of computational modeling. To computational biologists, negative data are as important as positive data, but negative observations are often neglected or discarded by experimental biologists without being collected in the public repositories. As a makeshift, computational biologists generally resort to random sampling to generate negative data. However, without experimental validation, randomly sampled data lack supporting biological evidence [13,14,15,16,17,18,19,20,21,22,23,24,25,26,27,28]. The evidence supporting random sampling merely comes from the theory of complex networks, which assumes PPI networks to be scale-free instead of random networks [29]. Under such an assumption, proteins do not interact randomly and thus two randomly sampled proteins do not interact with high probability. Though less biased, random sampling method runs a high risk of sampling false negative data and are hard to biologically interpret. To reduce the false negative rate, Ben-Hur et al. [30] selected those protein pairs that are not subcellularly co-localized as negative examples. As such, the obtained negative data are more reliable but less representative, because this method does not cover the subcellularly co-localized protein pairs that do not interact, which are more useful to reveal the PPI mechanisms. Blohm et al. [31] build the Negatome database to collect the experimentally verified or structurally curated non-interacting protein pairs. Unfortunately, Negatome is very small. Trabuco et al. [32] took advantage of the pairs of bait and prey observed via yeast two-hybrid technique to infer the bait-prey pairs that are not experimentally observed as negative, i.e., non-interaction. This policy is very smart but only applicable to two-hybrid experiments. Furthermore, the pairs of bait and prey are not reported to be simultaneously detected by the same experimental platform, thus the experimentally unobserved pairs of bait and prey are not necessarily negative.

*Model evaluation* is the fourth critical concern in computational or machine learning modeling. *K*-fold cross validation is generally a necessary step to evaluate model performance. Nevertheless, cross validation is not sufficient to check model bias, because exhaustive parameter tuning could in practice produce a seemingly perfect and unbiased model to fit the training data. What we are really concerned about is to gain knowledge about how well the model generalizes to unseen examples. For this reason, independent test is also an indispensable step to model evaluation. However, existing methods seldom conduct independent test or are merely evaluated on the positive independent test data [13,14,15,16,17,18,19,20,21,22,23,24,25,26,27,28]. The emphasis of evaluation on the negative independent test data is not because the negative results biologically matter but because we need to know how much the model is potentially biased. If a model performs very well on the positive independent test data but rather poorly on the negative independent test data, the model runs a high risk of bias toward the positive, so that the results are probably not so credible as to contain a high level of false positive predictions.

In this study, we use the very limited manually-curated negative data from Negatome as seeds to infer more negative data for computational modeling. The concept of interlog [33] assumes that the paralogs or orthologs of two interacting proteins also interact. Based on the notion of orthologous or paralogous structure conservation, we assume that the paralogs or orthologs of two non-interacting proteins also do not interact with high probability and coin this assumption as Neglog. To reduce the risk of bias toward the non-interactions from Negatome [31], we use the less biased random sampling method to enlarge the coverage of negative data. The positive training data are taken from the physical PPIs in HPRD [1] and BioGrid [2], and the positive independent test data are taken from Reactome [3], IntAct [5], and HitPredict [6]. The negative independent test data are taken from Negatome [31], random sampling, and Neglogs. The comprehensive estimation is to know about and reduce the risk of model bias. We adopt gene ontology (GO) terms as features and conduct homolog knowledge transfer to tackle GO sparsity. L_2_-regularized logistic regression is used as the predictive model to counteract noise from homologs and fast train on large dataset. Lastly, we validate the PPIs in STRING [7] using the proposed model and merge the validated PPIs into the comprehensive human physical PPI networks for further research. 

## 2. Materials and Methods

### 2.1. Data and Materials

#### 2.1.1. Positive Training Data

To ensure the data quality, we use the physical PPIs curated in HPRD [1] and BioGrid [2] as positive training data. These data all come from experimental observations. After removing those obsolete/uncurated proteins and the proteins that have no corresponding gene names in Uniprot (http://www.uniprot.org/uniprot/), we obtained 55,862 physical PPIs in total as the positive training data (see Supplementary file S1).

#### 2.1.2. Negative Training Data

The concept of interlog [33] assumes that the paralogs or orthologs of two interacting proteins also interact. Theoretically, two interacting proteins have their structures matched at the interface and thus their orthologs also probably have structures matched because of orthologous structure conservation. Based on the notion of paralogous or orthologous structure conservation, we assume that the paralogs or orthologs of two non-interacting proteins (coined as Neglog) also do not interact with high probability. We will validate and discuss the assumption in the Results section. Recently, homology modeling is being widely used in biological inference, e.g., functional annotation transfer between genes [33], protein structure prediction [34,35], etc. The studies [34,35] show that ortholog proteins are often structurally conserved even with a low sequence identity. Two proteins that hardly interact often have their structures spatiotemporally mismatched, so do their paralogs or orthologs because of paralogous or orthologous structure conservation. The chance that paralogs or orthologs of two non-interacting proteins would be much smaller than that of a random pair of proteins. Based on the notion “Neglog” we leverage the very limited experimentally verified negative data from Negatome [31] to infer more negative data for computational modeling.

For any two non-interacting proteins in Negatome [31] ¬(a,b), we obtain their corresponding human paralog set A,B, respectively. A Neglog derived from ¬(a,b) is as defined below.

(1) $${\mathrm{Neglog}}_{\neg (a,b)}=\{\neg (a,b)\}\cup \{\neg (a,b^{\prime })|b^{\prime }\in B\}\cup \{\neg (a^{\prime },b)|a^{\prime }\in A\}\cup \{\neg (a^{\prime },b^{\prime })|a^{\prime }\in A\wedge b^{\prime }\in B\}$$

The whole Neglog set is defined as follows.

(2) $$\mathrm{Neglog}=\underset{\neg (a,b)\in \mathrm{Negatome}}{\cup }{\mathrm{Neglog}}_{\neg (a,b)}$$

For two non-interacting proteins ¬(a,b), protein *a* and *b* are potentially structurally mismatched [31]. The paralog or ortholog $a^{\prime }$ and $b^{\prime }$ are structurally similar to protein *a* and *b*, such that $a^{\prime }$ and $b^{\prime }$ are more likely structurally mismatched than randomly sampled protein pairs. In this way, $\neg (a^{\prime },b^{\prime })$ could be inferred with high probability. Totally, we obtain 1027 manually-curated human protein pairs that do not interact with Negatome [31]. Using these protein pairs as seeds, we totally obtain 125,982 Neglogs with E-value equal to 1e-20 (see Supplementary file S2). As the Neglog set covers a very small number of genes/proteins in the Negatome database [31] and Neglog represents only one kind of protein–protein non-interactions, the negative data solely from Neglogs run a high risk of training a biased model. For these reasons, we resort to random sampling to enlarge the coverage of genes and meanwhile to reduce the risk of bias. The random sampling space is restricted within the well-studied human genes defined in SwissProt [36]. These human genes are annotated with at least one GO term of molecular function or biological process. The ratio of Neglog to random is 7:3 and the number of negative data is equal to that of positive data (see Supplementary file S3). Under the sampling ratio, the Neglog method introduces much fewer false negative data than the pure random sampling method. Human paralog genes are searched via PSI-BLAST [37] against SwissProt [36] with E-value equal to 1e-20.

#### 2.1.3. Independent Test Data

*K*-fold cross validation is a gold-standard method for model evaluation, whereas mere cross validation on training data is not sufficient because over-tuning of parameters in many cases could produce a seemingly perfect and unbiased model. The model probably shows a serious bias when further evaluated against independent test data. Independent test is an indispensable step to check how well a model generalizes to unseen examples or patterns. In this study, the positive independent test data are taken from Reactome [3], IntAct [5], and HitPredict [6]. For IntAct [5] and HitPredict [6], only human physical PPIs are chosen. As a result, we obtain 118,571 PPIs from Reactome [3], 1811 PPIs from IntAct [5], and 111,171 PPIs from HitPredict [6], respectively. The negative independent test data are sampled from three sources: (1) 200 non-interactions called *Experiments* are randomly sampled from Negatome [31]; (2) 118,571 protein pairs called *Random* are randomly sampled from the random space; and (3) 723,55 neglogs are randomly sampled from the Neglog space. We make sure that all the negative independent test data are disjoint with the training data and the positive independent test data.

#### 2.1.4. PPIs in STRING to Be Validated

STRING [7] has collected massive PPI networks of 2031 species, but most of the data are of low quality without being experimentally or computationally validated [34,35]. In this study, we use the proposed Neglog method to validate the human PPIs in STRING, based on which a comprehensive human physical PPI networks for further biomedical research is constructed.

### 2.2. Feature Construction

Gene ontology (GO) has been recognized to be the most discriminative feature to represent protein pairs or predict PPIs [26,27]. The GO terms are extracted from GOA [38]. For a less-studied gene, we transfer the GO knowledge of its homologs to enrich the feature information. Each gene/protein is depicted with two instances called target instance and homolog instance. The target instance represents the GO knowledge of the gene/protein itself, while the homolog instance represents the GO knowledge of the homologs. The homologs are queried against SwissProt [36] via PSI-BLast [37] and the GO terms are extracted from GOA [38]. The process of feature construction is formally described as follows.

For each protein *i* in the training set *U*, we obtain one target set of GO terms ($S_{T}^{i}$) and one homolog set of GO terms ($S_{H}^{i}$). The whole set of GO terms of the training set is defined as follows.

(3) $$S=\underset{i\in U}{\cup }(S_{T}^{i}\cup S_{H}^{i})$$

For a protein pair $\left(i_{1},i_{2}\right)$, the target instance and the homolog instance are formally defined as follows.

(4) $$V_{T}^{\left(i_{1},i_{2}\right)}[g]=\left\{ \begin{array}{l} 0,g\notin S_{T}^{i_{1}}\wedge g\notin S_{T}^{i_{2}}\\ 2,g\in S_{T}^{i_{1}}\wedge g\in S_{T}^{i_{2}}\\ 1,otherwise \end{array}\right.;\;V_{H}^{\left(i_{1},i_{2}\right)}[g]=\left\{ \begin{array}{l} 0,g\notin S_{H}^{i_{1}}\wedge g\notin S_{H}^{i_{2}}\\ 2,g\in S_{H}^{i_{1}}\wedge g\in S_{H}^{i_{2}}\\ 1,otherwise \end{array}\right.$$

For each GO term g∈S, $V_{T}^{\left(i_{1},i_{2}\right)}[g]$, and $V_{H}^{\left(i_{1},i_{2}\right)}[g]$ denote the component *g* of the target instance and the homolog instance, respectively. Those GO terms g∉S are discarded. Formula (4) means that if the protein pair $\left(i_{1},i_{2}\right)$ shares the same *GO* term *g*, then the corresponding component in the target and homolog feature vectors is set to 2; if neither protein in the protein pair possesses the *GO* term *g,* then the component is set to 0; otherwise the component is set to 1.

### 2.3. L_2_-regularized Logistic Regression as Base Classifier

Homolog knowledge transfer can well tackle GO sparsity but it also introduces noise from evolutionary divergence. Regularization technique is a commonly-used policy to penalize noise and reduce the risk of overfitting, e.g., SVM [39]. However, the kernel method SVM with computational complexity $O(n^{2})$ is not well scaled to a large training set. In this study, homolog knowledge transfer helps to tackle GO sparsity but at the same time increases the computational complexity by two-fold. To counteract homolog noise and fit large training data in linear time, l_2_-regularized logistic regression method [40], implemented in the toolbox LIBLINEAR [41], is adopted as the base classifier.

Given a set of instance-label pairs $\left(x_{i},y_{i}),i=1,2,\ldots ,l;x_{i}\in R^{n};y_{i}\in \{-1,+1\right\}$, l_2_-regularized logistic regression solves the following optimization problem.

(5) $$\underset{\omega }{\min}\frac{1}{2}\omega^{T}\omega +C\sum_{i=1}^{l}\log(1+e^{-y_{i}\omega^{T}x_{i}})$$

where ω denotes the weight vector and C denotes the penalty parameter or regularizer. The second term is designed to penalize potential noise/outlier fitting. The optimization of objective function (5) is solved via its dual form.

(6) $$\begin{array}{l} \underset{\alpha }{\min}\frac{1}{2}\alpha^{T}Q\alpha +\sum_{i:\alpha_{i}>0}^{l}\alpha_{i}\log\alpha_{i}+\sum_{i:\alpha_{i}<C}\left(C-\alpha_{i}\right)\log(C-\alpha_{i})-\sum_{i}^{l}C\log C\\ s.t.\;0\leq \alpha_{i}\leq C,i=1,\ldots ,l \end{array}$$

where $\alpha_{i}$ denotes Lagrangian operator and $Q_{ij}=y_{i}y_{j}x_{i}^{T}x_{j}$.

For each test protein pair $\left(i_{1},i_{2}\right)$, the decision function f(x) yields two outputs for the target instance ($f(V_{T}^{\left(i_{1},i_{2}\right)})$) and the homolog instance ($f(V_{H}^{\left(i_{1},i_{2}\right)})$), respectively. The two outputs are further combined into a final decision value as follows.

(7) $$Decision\_value(i_{1},i_{2})=\left\{ \begin{array}{l} f(V_{T}^{\left(i_{1},i_{2}\right)}),if|f(V_{T}^{\left(i_{1},i_{2}\right)})|>|f(V_{H}^{\left(i_{1},i_{2}\right)})|\\ f(V_{H}^{\left(i_{1},i_{2}\right)}),otherwise \end{array}\right.$$

where |•| denotes the absolute value The final label for the test protein pair $\left(i_{1},i_{2}\right)$ is defined as follows.

(8) $$L(i_{1},i_{2})\pm =\left\{ \begin{array}{l} 1,Decision\_value(i_{1},i_{2})>0\wedge Decision\_value(i_{1},i_{2})-0.5>\delta \\ -1,Decision\_value(i_{1},i_{2})<0\wedge -Decision\_value(i_{1},i_{2})-0.5>\delta \\ \propto ,otherwise \end{array}\right.$$

where the threshold δ is used to filter out the weak positive predictions, ∝ denotes the undetermined predictions, which will be discarded. Formula (8) means that only the predictions that are δ larger than random guess are accepted.

### 2.4. Experimental Setting and Model Evaluation

To verify the effectiveness of homolog knowledge transfer, we compare its performance under the three experimental settings, namely combine-instance, homolog-instance, and target-instance. The combined-instance setting combines the outputs of target instance and homolog instance. The homolog-instance setting uses the homolog instance alone to deliberately evaluate the robustness against GO sparsity or unavailability. The target-instance setting uses the target instance alone to estimate the baseline performance. Equivalence to or excellence over the baseline performance indicates that homolog knowledge transfer is effective.

The proposed model is evaluated by five performance metrics including receiver operating characteristic AUC (ROC-AUC), sensitivity (SE) or recall (RE), precision (PR), Matthews correlation coefficient (MCC), and F1 score. The predicted results of *k*-fold cross validation are stored in a confusion matrix *M*, each element of which $M_{i,j}$ records the counts that class i are classified to class j. Several intermediate variables are calculated on the basis of *M* according to Formula (9). Based on these intermediate variables, PR_l_, SE_l_, and MCC_l_ for each label are calculated according to Formula (10). The overall MCC is calculated via Formula (11).

(9) $$\begin{array}{l} p_{l}=M_{l,l},q_{l}=\sum_{i=1,i\neq l}^{L}\sum_{j=1,j\neq l}^{L}M_{i,j},r_{l}=\sum_{i=1,i\neq l}^{L}M_{i,l},s_{l}=\sum_{j=1,j\neq l}^{L}M_{l,j}\\ p=\sum_{l=1}^{L}p_{l},q=\sum_{l=1}^{L}q_{l},r=\sum_{l=1}^{L}r_{l},s=\sum_{l=1}^{L}s_{l} \end{array}$$

(10) PRl=plpl+rl,l=1,2…,LSEl=plpl+sl,l=1,2…,LMCCl=(plql−rlsl)/(pl+rl)(pl+sl)(ql+rl)(ql+sl),l=1,2…,L

(11) $$\begin{array}{l} Acc=\sum_{l=1}^{L}M_{l,l}/\sum_{i=1}^{L}\sum_{j=1}^{L}M_{i,j}\\ MCC=\left(pq-rs\right)/\sqrt{\left(p+r)(p+s)(q+r)(q+s\right)} \end{array}$$

where *L* denotes the number of labels. In this study, PPI prediction is a problem of binary classification (*L* = 2). The metrics PR_l_, SE_l_, and MCC_l_ on each class are calculated to estimate the potential model bias. AUC is calculated on the basis of decision values as defined in Formula (7). F1 score is defined as follows.

(12) F1 score=2×PRl×SElPRl+SEl,l= 1 denotes the positive class

## 3. Results

### 3.1. Validating the Assumption of Neglog 

At present, Negatome [31] manually curates 1027 human protein pairs that are experimentally observed not to interact. Among the protein pairs, there are no paralogous or orthologous relationships, rendering it difficult to validate the Neglog assumption directly through experimental data. For this reason, we take the protein pair (PKN1, RPS6KA1) for example to validate the assumption of Neglog from two aspects, i.e., protein structure alignment and functional validation.

#### 3.1.1. Protein Structure Alignment

**Figure 1:**
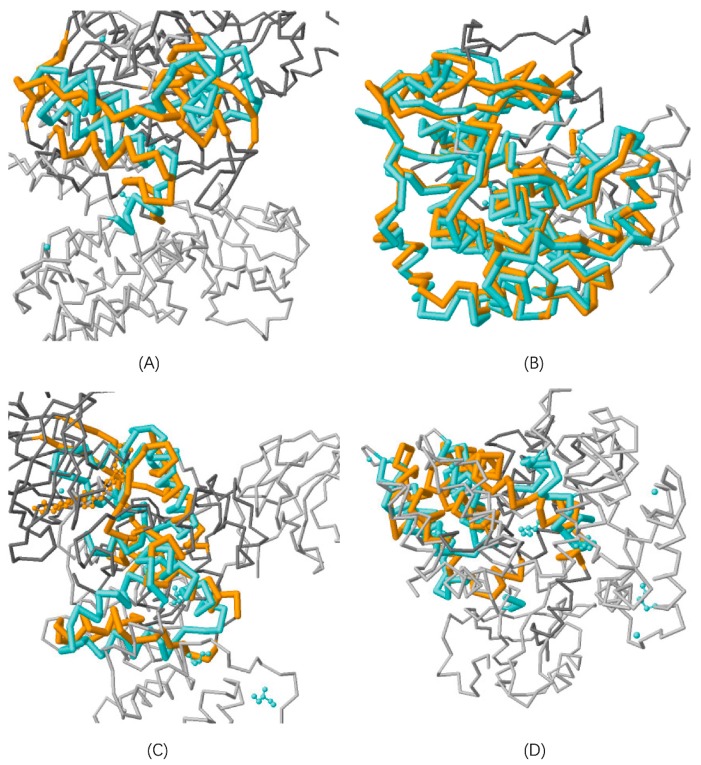
Paralog structural comparisons of two non-interacting proteins. (**A**) illustrates the structural alignment between protein pair (PKN1, RPS6KA1) that do not interact with Z-score equal to 1.99, identity equal to 2.75%, and similarity equal to 9.89%; (**B**) illustrates the structural alignment between paralog (PKN1, AKT1) with Z-score equal to 6.35, identity equal to 39.00%, and similarity equal to 55.67%; (**C**) illustrates the structural alignment between Neglog (AKT1, RPS6KA1) with Z-score equal to 1.99, identity equal to 4.35%, and similarity equal to 10.33%; (**D**) illustrates the structural alignment between a random protein BAD and RPS6KA1 (BAD, RPS6KA1) with Z-score equal to 3.70, identity equal to 4.38%, and similarity equal to 13.14%.

From structural point of view, two interacting proteins possess shape complementarity or secondary structural similarity between the interface surfaces [42,43]. The structural conservation of orthologs helps reserve the structure of interacting interface between orthologs, leading to the assumption of Interlog. Between two proteins that do not interact, there may not exist shape complementarity or structural similarity to form an interacting interface. Taking the protein pair (PKN1, RPS6KA1) from Negatome [31] for example, protein structure alignment [44] (http://www.rcsb.org/pdb/workbench/workbench.do) shows that PKN1 and RPS6KA1 are structurally dissimilar with Z-score equal to 1.99, identity equal to 2.75%, and similarity equal to 9.89% 2.30 (see Figure 1A). We choose AKT1 as one paralog of PKN1. Protein structure alignment shows that AKT1 and PKN1 are structurally similar with Z-score equal to 6.35, identity equal to 39.00%, and similarity equal to 55.67% (see Figure 1B). According to Equations (1 and 2), (AKT1, RPS6KA1) is one Neglog inferred from the non-interacting protein pair (PKN1, RPS6KA1). Protein structure alignment shows that the alignment level of the Neglog (AKT1, RPS6KA1) is equivalent to that of (PKN1, RPS6KA1), with Z-score equal to 1.99, identity equal to 4.35%, and similarity equal to 10.33% (see Figure 1C). We can infer that the paralog pair (AKT1, PKN1) does not have their core structures varied much. These results show that the structural mismatch between PKN1 and RPS6KA1 is conserved across the Neglog (AKT1, RPS6KA1) via paralogous structure conservation between PKN1 and AKT1. For the reason, the Neglog (AKT1, RPS6KA1) is assumed not to interact.

To further validate the assumption of Neglog, we randomly sample a protein BAD (not orthologous to PKN1) from the random space to check its structural mismatch with protein RPS6KA1. Protein structure alignment shows that the alignment level of the random protein pair (BAD, RPS6KA1) is much larger than the Neglog (AKT1, RPS6KA1) with Z-score equal to 3.70, identity equal to 4.38%, and similarity equal to 13.14% (see Figure 1D). This result shows that the Neglog inferred from Negatome more unlikely interacts than randomly sampled protein pairs.

#### 3.1.2. Functional Validation

Functional analyses show that protein PKN1and its paralog protein AKT1 get involved in fairly similar cellular processes. PKN1 is PKC-related serine/threonine-protein kinase that is involved in various processes, e.g., regulation of the intermediate filaments of the actin cytoskeleton, cell migration, tumor cell invasion, and transcription regulation, etc., (https://www.uniprot.org/uniprot/Q16512). The paralog protein AKT1 possesses more divergent functions but still participates in several core processes shared with PKN1, e.g., regulation of cell migration (GO:0030334), negative regulation of protein kinase activity by protein phosphorylation (GO:0100002), cellular response to tumor necrosis factor (GO:0071356), positive regulation of transcription by RNA polymerase II (GO:0045944), etc., (https://www.uniprot.org/uniprot/P31749). The core processes shared between PKN1 and its paralog AKT1 may potentially indicate that AKT1 also do not interact with RPS6KA1.

### 3.2. Performance of Cross Validation and Independent Test

#### 3.2.1. Cross Validation 

**Figure 2:**
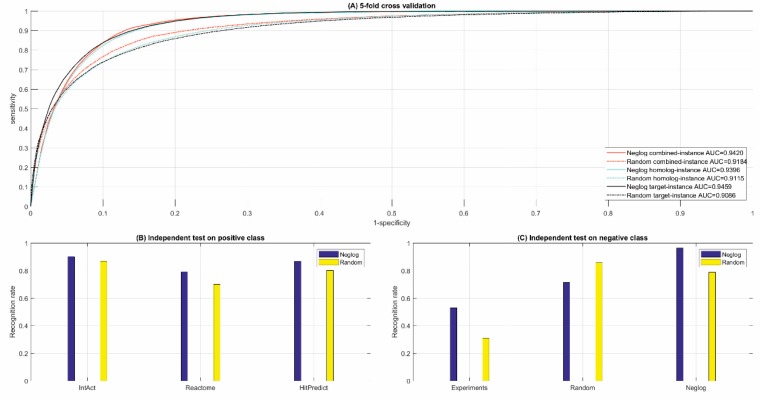
Performance of cross validation and independent test.

The ROC curves of five-fold cross validation are illustrated in Figure 2A and the results regarding other performance metrics are summarized in Table 1. The Neglog method achieves equivalently high AUC scores for the three settings, e.g., 0.9420 for the combine-instance setting. The other performance metrics also show that the Neglog method achieves equivalently encouraging performance under the three settings, indicating that homolog knowledge transfer is effective to tackle GO sparsity for the less-studied genes. Further comparison shows that the Neglog method is little biased toward the positive class, e.g., 0.9154 and 0.8568. Recall rate on the positive and negative class in the combine-instance setting (see Table 1), respectively. The bias was partly due to the lack of experimentally observed negative data.

#### 3.2.2. Independent Test 

*Positive independent test.* As shown in Table 1, the Neglog method demonstrates fairly good performance on the positive independent test data, e.g., 90.20% and 86.76% recognition/recall rate on IntAct and HitPredict, respectively. The performance of 79.14% recognition/recall rate on Reactome is still acceptable. The smallest independent test data from IntAct [5] (1811 PPIs) achieve the best recall/recognition rate and the large independent test data from Reactome [3] (118,571 PPIs) and HitPredict [6] (111,171 PPIs) achieve relatively poor recognition rate. It seems that the predictive performance deteriorates with the size of independent test data. Actually, the size of independent test data hardly affects the performance of a credible and robust predictive model. The reason is majorly because Reactome database contains a large number of functional PPIs [45] and the HitPredict database also contains predicted PPIs [6], whereas the positive training data of the proposed Neglog method are all physical PPIs. The independent test data from IntAct [5] are also physical PPIs and thus demonstrate high performance. 

*Negative independent test.* Independent test on negative data is easily ignored in computational or machine learning modeling for biological problems partly because biologists focus on positive results. However, the performance of independent test on negative class is significant for computational biologists to know whether or how much the model is biased. As shown in Table 1, the Neglog method correctly recognizes 96.40% of the independent test Neglogs, indicating that the model is not obviously biased toward the positive class. Because of the low ratio of Random to Neglog (3:7), the Neglog method only achieves 71.38% recognition/recall rate on randomly sampled independent test data. On the negative independent test data from experiments, the Neglog method only achieves 53.00% recognition/recall rate. Although the Neglog method embraces a large number of quality positive data (55,862 physical PPIs), the small number of experimental negative data and Neglog inference from these seeds still affects the model to generalize unseen negative examples. Unfortunately, the existing methods generally ignores the importance of independent test on negative class.

This result also shows that a model demonstrating good and unbiased performance of cross validation on the training data does not necessarily promise to generalize well to unseen examples. The risk of bias needs further evaluation via independent test. Although the performance on the negative independent test data from experiments is not satisfactory, we found that the random sampling method that contain no negative training data from experiments performs much worse (see the next section *Comparison with random sampling*). From computational point of view, negative data are as important as positive data. That is why we develop the Neglog method to exploit and augment the available experimental negative data in this study. For further biomedical research, the validated PPIs in IntAct, Reactome, and HitPredict are merged into Supplementary file S4 and the remaining PPIs are merged into Supplementary file S5. The protein pairs in the negative independent test data validated by the Neglog method are merged into Supplementary file S6. 

### 3.3. Comparison with Random Sampling

#### 3.3.1. Cross Validation

As shown in Figure 2A,B and Table 1 and Table 2, random sampling method underperforms the Neglog method in terms of all metrics, e.g., 6% decrease of MCC scores. Altogether, the performance decrease is not large and still acceptable. These results show that random sampling, as a commonly-used method, is still a good solution to computational modeling for biological problems when the required experimental negative data are not available. Actually, the Neglog method also obtains a small number of protein pairs as negative data via random sampling to reduce the risk of data bias.

#### 3.3.2. Independent Test 

As shown in Figure 2C, and Table 1 and Table 2, the Neglog method obviously achieves far better performance than random sampling method, e.g., 79.14% versus 70.02% recognition/recall rate on Reactome, 86.76% versus 80.20% recognition/recall rate on HitPredict. Especially on the experimental negative independent test data, the Neglog method achieves 53.00% recognition/recall rate while random sampling method only achieves 31.00% recognition/recall rate. The large performance difference demonstrates the significance of good-quality negative data to computational modeling. On the Neglog independent test data, the Neglog method also performs much better than random sampling method with 96.40% versus 78.74% recognition/recall rate. But on the random independent test data, the Neglog method performs much worse than random sampling method with 71.38% versus 85.85% recognition/recall rate, which partly results from the low ratio of Random to Neglog (3:7) in the negative training data.

In summary, the Neglog method generally performs better than random sampling in terms of cross validation and independent test. Especially, the Neglog method recognizes the PPIs from Reactome [3], IntAct [5], and HitPredict [6] with higher accuracy than random sampling method. Furthermore, the Neglog method outperforms the random sampling method with 53.00% versus 31.00% recognition/recall rate on the negative independent test data from experiments. Besides performance, the Neglog method possesses better biological interpretability than random sampling method in that the Neglog assumption is biologically based on orthologous/paralogous structure conservation. Nevertheless, the Neglog method potentially demonstrates a larger bias than random sampling method. With the accumulation of experimentally observed negative data, the bias can be reduced.

### 3.4. Comparison with Existing Methods

#### 3.4.1. Subcellularly Restricted Sampling Methods

Two proteins physically separated into different cellular compartments have less chance to interact with each other. Based on this intuitive observation, subcellularly restricted sampling method [30] randomly samples protein pairs as negative data from the space of protein pairs that are not subcellularly co-localized. This method can yield reliable negative data but is highly biased, because it does not cover the proteins that are subcellularly co-localized but do not interact. Accordingly, the subcellularly restricted sampling method yields 0.83 ROC-AUC score [30]. 

Sun et al. [46] also adopted subcellularly restricted sampling method to generate negative data. The difference is that Sun et al. used *k*-mer as a feature and used deep learning as a base learner. Sun et al. reported 99.21% training accuracy on HPRD and 87.99–99.21% accuracy on several external datasets, i.e., positive independent test data. Training accuracy is usually used to estimates the fitness of training data but not to estimate how well a model generalizes to unseen examples. Furthermore, the method does not report the metrics of SE and PR on the negative class, so that we have no knowledge about the model bias. As a result, the model achieves 94.34% accuracy on a positive independent data but only 6.7% accuracy on a negative independent data. These results show that the model [46] runs a high risk of overtraining/overfitting and bias.

The Neglog method comprehensively reports its performance of cross validation and independent test on both positive and negative class. The results show that the Neglog method is also a bit biased toward the genes/proteins in Negatome, but it combines random sampling to reduce bias with ROC-AUC score up to 0.9420 for the combined-instance setting. The accuracies on the positive independent test data range from 79.14% to 90.20%. Even the worst accuracy on the negative independent test data is up to 53.00%.

#### 3.4.2. Sequence Feature Based Methods

Sequence features such as *k*-mer have been intensively used in biological sequence analyses including PPI prediction [13,14,15,45,46] because of the easy availability. Nevertheless, sequence feature is restricted in practical applications because of its capability of predicting protein–protein interactions, averagely 0.72 ROC-AUC score of cross validation on HPRD [14,15]. This performance is not encouraging.

The existing methods seldom report their performance on the negative class and performance of independent test, such that we have no knowledge about the risk of model bias. Park et al. [15] randomly sampled negative data with the ratio of positive to negative equal to 1:2000. Such a highly skewed class distribution takes a high risk of bias. Actually, biological space cannot be directly mapped onto computational space. A highly biased model is actually useless. Even so, we have no idea about the true ratio of positive to negative class in PPI space.

#### 3.4.3. GO Feature Based Methods

Maetschke et al. [27] used GO term as a feature and demonstrated the excellence of GO terms over sequence features in predicting protein–protein interactions. Unfortunately, the authors did not report the performance of cross validation and independent test on human dataset. They only reported 0.85 AUC score of self-test, i.e., training and test are conducted on the same dataset, which is actually training accuracy. Furthermore, the cross-species AUC score between *H. sapiens* and *E. coli*, i.e., training on human data, and testing on *E. coli* data, is up to 0.87 and is surprisingly larger than 0.85 self-test AUC score. The genome gap between *H. sapiens* and *E. coli* makes it hard to interpret this result. In addition, AUC score alone is not sufficient to estimate the model performance, especially model bias. The Neglog method and random sampling method both achieved high AUC scores of cross validation but at the same time both showed bias, especially on the negative independent test data from experiments (53.00% recall rate).

Maetschke et al. [27] incorporated related GO terms via common ancestor, shortest path and semantic similarity of GO terms in GO directed acyclic graph (DAG). The method could well tackle GO sparsity and enrich feature information but at the same time introduced inter-feature correlation, which potentially deteriorated the model performance. In the Neglog method, simple flat feature representation is used to depict the co-occurrence and individual presence or absence of GO terms in a pair of proteins. We do not consider the hierarchical organization or semantic relationships in GO DAG to minimize inter-feature correlations. To tackle GO sparsity, we use an additional homolog instance to borrow the GO terms of homologs.

### 3.5. Validation of Human Protein-Protein Interactions in STRING [7]

#### 3.5.1. Statistics of PPI Validation

There are more than 4 million human protein–protein interactions in STRING [7] to date, which is far beyond biologists’ estimation. Recent studies also report that the PPI data in STRING [7] are of low quality [23,24]. To reduce computational complexity, we only choose the PPIs with evidence of experiments, gene co-expression, database, gene fusion, co-occurrence, and text mining. For the last three evidences, we exert individual constraints of score threshold ≥200, ≥800, and ≥200, respectively. All the PPIs extracted from STRING [7] are disjoint with the training data and the independent test data. The data distribution and the positive rates of different evidence predicted by the Neglog method are provided in Table 3. The Neglog method validates 379,782 PPIs (see Supplementary file S7) and the remaining 180,849 PPIs are provided in Supplementary file S8. The total predicted positive rate is relatively high. To reduce the risk of false positive predictions, a higher threshold δ as defined in Equation (8) is recommended.

As shown in Table 3, the Neglog method achieves positive rate of 81.69 %, 77.41 %, and 71.09% on the evidence of *experiments*, *database,* and *text mining*, respectively, much higher than those on the other indirect evidence including co-expression, fusion, co-occurrence, and text mining. 

#### 3.5.2. Case Study on YBX2

**Figure 3:**
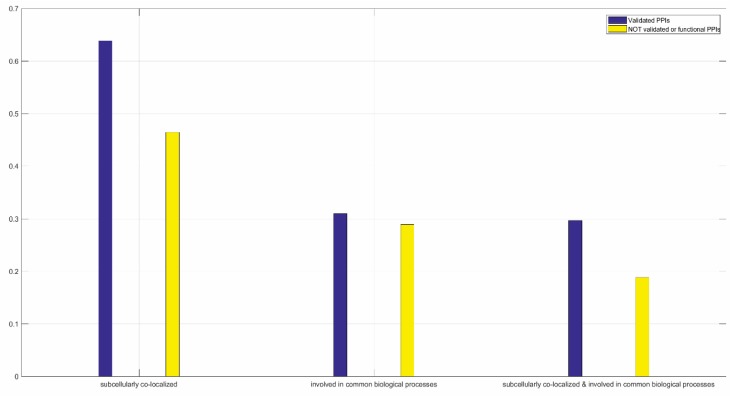
Statistical analyses of subcellular co-localization and involvement in common biological processes of the partners that are validated and NOT validated to interact with the protein YBX2. The PPIs are extracted via the evidence of co-expression in STRING.

Taking the protein YBX2 for example, we extract 394 PPIs from STRING that are inferred from the evidence of *co-expression*. The Neglog validates 155 PPIs and still predicts the remaining 239 PPIs as negative. As regards the validated and the not validated PPIs, we analyze the statistics of subcellular co-localization common biological processes as illustrated in Figure 3. The statistics show that the PPIs validated by the Neglog method tend to subcellularly co-localize and get involved in common biological processes than the PPIs that are not validated by the Neglog method. 

**Figure 4:**
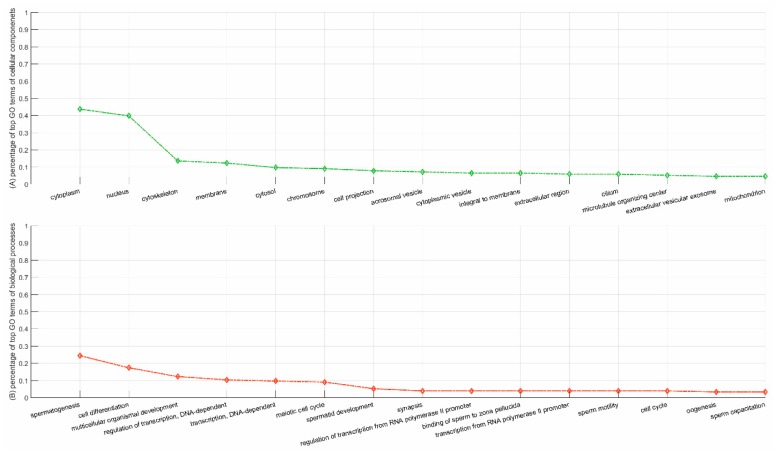
Percentages of top gene ontology (GO) terms of the genes/proteins that are validated to interact with the protein YBX2. The protein–protein interactions (PPIs) are extracted via the evidence of co-expression in STRING. (**A**) illustrates the percentage of top GO terms of cellular components; and (**B**) illustrates the percentage of top GO terms of biological processes.

The protein YBX2, mainly subcellularly localized in nucleus (GO:0005634) and cytoplasm (GO:0005737), majorly gets involved in the cellular processes of regulation of the stability and/or translation of germ cell mRNAs (https://www.uniprot.org/uniprot/Q9Y2T7), e.g., spermatogenesis (GO:0007283), translational attenuation (GO:0009386), regulation of transcription, DNA-templated (GO:0006355), etc. The top 15 GO terms of YBX2 partners are illustrated in Figure 4. Similar to YBX2, the partners are also primarily subcellularly localized in nucleus (GO:0005634), cytoplasm (GO:0005737), cytoskeleton (GO:0005856), chromosome (GO:0000775), cilium (GO:0005929), etc. Meanwhile, these partners get involved in similar biological processes as the protein YBX2, e.g., spermatogenesis (GO:0007283), cell differentiation (GO:0030154), meiotic cell cycle (GO:0051321), spermatid development (GO:0007286), sperm motility (GO:0030317), etc. GO enrichment analyses to a certain degree validates the rationality of the predictions of the Neglog method.

**Figure 5:**
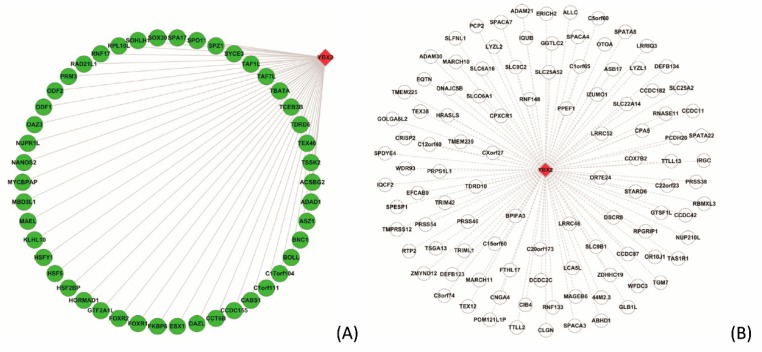
The validated and not validated PPIs related to the protein YBX2. The PPIs are extracted via the evidence of co-expression in STRING. (**A**) illustrates the PPIs that are validated by the proposed Neglog method. Only the PPIs whose partners are subcellularly colocalized and get involved common biological processes are illustrated for clarity; (**B**) illustrates the PPIs that are NOT validated, and only the partners that are not subcellularly co-localized and not involved in common biological processes are illustrated for clarity. The nodes in green connected with solid lines indicate the partners that interact with the protein YBX2, while the nodes in white connected with long dash lines indicate the proteins that do not interact or functionally interact with the protein YBX2.

The PPIs related to YBX2 that are validated and not validated by the Neglog method are illustrated in Figure 5A,B, respectively. For clarity purpose, only the PPIs whose partners are subcellularly colocalized and get involved common biological processes are illustrated in Figure 5A. Similarly, only the partners that are not subcellularly co-localized and not involved in common biological processes are illustrated in Figure 5B. Taking the interaction between YBX2 and ASZ1 that is validated by the Neglog method for example, the partner ASZ1 is mainly localized in cytoplasm (GO:0005737) and plays a central role in spermatogenesis via repressing transposable elements and preventing their mobilization (https://www.uniprot.org/uniprot/Q8WWH4). ASZ1 is annotated with the GO terms including spermatogenesis (GO:0007283), cell differentiation (GO:0030154), meiotic cell cycle (GO:0051321), male meiotic nuclear division (GO:0007140), etc. These GO terms are similar to the cellular processes that YBX2 gets involved in. 

For PPIs from STRING [7] that are not validated by the Neglog method, we take the interaction between YBX2 and GGTLC2 for example. The protein GGTLC2 is localized in extracellular exosome (GO:0070062) and gets involved in glutathione catabolic process (GO:0006751), leukotriene D4 biosynthetic process (GO:1901750), etc (https://www.uniprot.org/uniprot/Q14390). These GO terms suggest that GGTLC2 is less related to the cellular processes that YBX2 gets involved in.

## 4. Discussion

Protein-protein interaction (PPI) networks play important roles in inferring signaling pathways, discovering network hallmarks of disease, screening drug targets and estimating pharmacological risks. Recent years have witnessed much progress in computational reconstruction of protein–protein interaction networks. Nevertheless, the existing computational methods still leave several major issues to be properly addressed. In this study, we comprehensively review the existing methods from the aspects of (1) data quality; (2) feature construction; (3) negative data sampling; (4) and model evaluation. Based on these issues, we focus on how to sample credible and biologically interpretable negative data for genome-scale reconstruction of human protein–protein interaction networks.

Existing methods as a whole pay more attention to feature extraction or developing/applying novel algorithms (e.g., deep learning). Although the predictive performance has been greatly improved, the improvement brought about by novel methods is very limited. The issue of negative data sampling potentially affects the model performance heavily, but it has received little attention in recent years. Random sampling is less biased and easily hits protein pairs that do not interact in scale-free PPI networks. As such, it is still the major solution adopted by the existing methods. However, random sampling is by nature hard to interpret biologically and potentially yield a certain level of false negatives. To reduce the risk of false negative sampling, subcellularly restricted random sampling sample negative data from the space of protein pairs that are not subcellularly co-localized. Though more credible, the method are highly biased because it does no cover the protein pairs that are subcellularly co-localized but do not interact.

In this study, we propose the assumption of Neglog to exploit and augment the available experimental negative data from Negatome [31] for genome-scale reconstruction of human protein–protein interaction networks. The assumption is based on structural conservation between a protein and its ortholog/paralog protein. Two proteins that do not interact are potentially mismatched spatiotemporally, which also probably take place between orthologs/paralogs. Because of the limited coverage of genes in Negatome, it is hard to validate the assumption via Negatome and we resort to protein structure alignment for analyses. Protein structure alignments show that the orthologs/paralogs of two interacting proteins are more structurally mismatched than a randomly sampled protein pair. As compared with random sampling, the Neglog method is easily interpretable and more credible. As compared with orthologs, paralogs develop new functions and have their structures varied, so that the paralogs of two proteins that do not interact still could develop a chance to interact. This noise could be to some extent counteracted by L_2_-regualrized logistic regression model.

As the Negatome database only covers a very small number of genes/proteins, the Neglog set derived from this seed still covers very limited genes. As a result, the negative data solely from Neglogs run a high risk of training a biased model. To our knowledge, there are no other sources that manually curate a large number of human genes that do not interact. To enlarge the negative class space and reduce bias, the Neglog method also uses random sampling to sample a certain ratio of negative data. As the ratio of randomly sampled negative data is very low (equal to 0.3), the risk of introducing false negative data is much reduced. Reducing positive training data to the size of negative data in Negatome to train an ensemble of individual classifiers may be another alternative solution. This solution could reduce the bias of each individual classifier. However, as all the individual classifiers are trained on the same negative data, the diversities of individual classifiers are very limited. As a result, the final ensemble classifier trained in the small space of negative data is still potentially highly biased.

Cross validation and independent test show that the Neglog method achieves much better and less biased performance than random sampling. In addition, the Neglog methods outperforms existing methods in terms of ROC-AUC scores. Computational results show that ROC-AUC score is not sufficient to estimate the potential model bias and independent test especially on the negative class is indispensable for model evaluation. Lastly, we use the trained model to validate the PPIs from STRING [7], in which most PPIs are not experimentally or computationally validated. GO enrichment analyses of the predicted partners of protein YBX2 to some extent validates the rationality of predictions. 

## Figures and Tables

**Table 1:** Netlog method performance of five-fold cross validation and independent test.

Cross Validation.	Size	Combined-Instance				Homolog-Instance				Target-Instance			
		PR	SE		MCC	PR	SE		MCC	PR	SE		MCC
Positive class	55,862	0.8653	0.9154		0.7957	0.8641	0.8944		0.7786	0.8617	0.9112		0.7864
Negative class	55,862	0.9098	0.8568		0.7934	0.8896	0.8582		0.7767	0.9018	0.8480		0.7823
[Acc; MCC; ROC-AUC]		[88.62%; 0.7935; 0.9420]				[87.64%; 0.7773; 0.9396]				[87.64%; 0.7773; 0.9459]			
F1 Score		0.8897				0.8790				0.8858			
Independent test		Positive class *						Negative class *					
		IntAct		Reactome		HitPredict		Experimental		Random		Neglog	
		90.20%		79.14%		86.76%		53.00%		71.38%		96.40%	

* denotes recall or recognition rate.

**Table 2:** Random sampling method performance of five-fold cross validation and independent test.

Cross Validation	Size	Combined-Instance				Homolog-Instance				Target-Instance			
		PR	SE		MCC	PR	SE		MCC	PR	SE		MCC
Positive class	55,862	0.8366	0.8726		0.7368	0.8269	0.8544		0.7137	0.8168	0.8714		0.7086
Negative class	55,862	0.8647	0.8269		0.7332	0.8443	0.8154		0.7090	0.8454	0.7825		0.6951
[Acc; MCC; ROC-AUC]		[85.00%; 0.7345;0.9184]				[83.52%; 0.7113;0.9115]				[83.52%; 0.7113;0.9086]			
F1 Score		0.8542				0.8404				0.8432			
Independent test		Positive class *						Negative class *					
		IntAct		Reactome		HitPredict		Experimental		Random		Neglog	
		86.99%		70.02%		80.20%		31.00%		85.85%		78.74%	

* denotes recall or recognition rate.

**Table 3:** Validation of human protein–protein interactions in STRING [7].

	Experiments	co-Expression	Database	Fusion	co-Occurrence	Text Mining
**Size**	38,323	148,227	93,225	987	11,435	289,826
**Positive Rate**	81.69 %	54.23 %	77.41 %	50.05 %	46.01 %	71.09%

## Supplementary Materials

Supplementary materials can be found at https://www.mdpi.com/1422-0067/20/20/5075/s1.
